# A thirty-three gene-based signature predicts lymph node metastasis and prognosis in patients with gastric cancer

**DOI:** 10.1016/j.heliyon.2023.e17017

**Published:** 2023-06-05

**Authors:** Jian Xiao, Gang Wang, Chuming Zhu, Kanghui Liu, Yuanhang Wang, Kuan Shen, Hao Fan, Xiang Ma, Zekuan Xu, Li Yang

**Affiliations:** a Department of General Surgery, The First Affiliated Hospital of Nanjing Medical University, Nanjing, Jiangsu Province, China; b Department of General Surgery, Liyang People's Hospital, Liyang Branch Hospital of Jiangsu Province Hospital, Liyang, Jiangsu Province, China

**Keywords:** Gastric cancer, Lymph node metastasis, Prognosis, Gene signature

## Abstract

Recently, several studies have indicated the great potential of gene expression signature of the primary tumor in predicting lymph node metastasis; however, few current gene biomarkers can predict lymph node status and prognosis in gastric cancer (GC). Thus, we used the RNA-seq data from The Cancer Genome Atlas (TCGA) to identify differentially expressed genes between pathological lymph node-negative (pN0) and positive (pN+) patients and to establish a gene signature that could predict lymph node metastasis. Meanwhile, the robustness of identified gene signatures was validated in an independent dataset Asian Cancer Research Group (n = 300). In this study, our thirty-three gene-based signature was highly correlated with lymph node metastasis and could successfully discriminate pN + patients in the training set (Area under the receiver operating characteristic curve = 0.951). Moreover, Disease-free survival (*P* = 0.0029) and overall survival (*P* = 0.026) were significantly worse in high-risk compared with low-risk patients overall and when confined to pN0 patients only (*P* < 0.0001). Of note, this gene signature also proved useful in predicting lymph node status and survival in the validation cohort. The present study suggests a thirty-three gene-based signature that could effectively predict lymph node metastasis and prognosis in GC.

## 1 Introduction

Lymph node status is vital for treatment decisions and outcome prediction of gastric cancer (GC) [1,2]. However, current methods for nodal metastasis prediction are largely dependent on preoperative imaging examinations [3] or pathological characteristics [4], which are not very sensitive and are inadequate [5]. Notably, the ability of tumor cells to metastasize is mainly dictated by the biological property of the tumor [6]. Thus, understanding the genomic differences between pathological lymph node-negative (pN0) and positive (pN+) patients is essential for predicting lymph node metastasis (LNM) and personalized treatment decisions.

In recent years, numerous studies have confirmed the great potential of gene markers in the risk stratification and prognostic prediction of tumors [[7], [8], [9]]. For example, a scoring system based on the expression of six genes successfully predicted the relapse of gastric cancer patients after curative resection [10]. Meanwhile, a study in lung adenocarcinomas suggested that a 318-gene set could predict lymph node status and even occult disease [11]. However, an effective gene signature-based scoring system for prognosticating LNM in GC is still lacking [12,13]. In addition, evaluation of gene expression signature is a reliable way to identify patients at risk for occult disease and worse survival. Hence, the goals of this study were to determine whether analysis of gene expression in GC could predict LNM and if so, predict future recurrence.

In this study, we used the RNA-seq data of The Cancer Genome Atlas (TCGA) to identify a gene signature that can predict LNM and recurrence in GC. Furthermore, microarray data from the Asian Cancer Research Group (ACRG) was introduced as a validation set.

## 2 Material and methods

### 2.1 Gene expression data acquisition

**Fig. 1:**
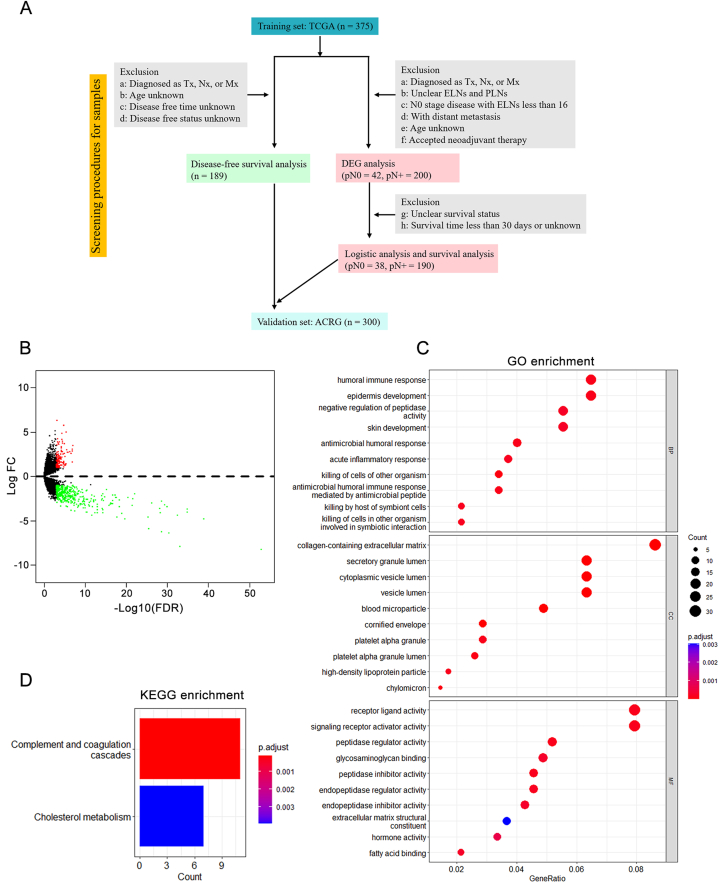
Identification of differentially expressed genes between pN0 and pN + samples. **A** Flow chart of the screening procedures for applicable samples. **B** The volcano plot shows differentially expressed genes in the TCGA cohort. Red dots represent upregulated genes, and green dots reveals downregulated genes. **C**GO terms enriched in DEGs. **D** KEGG pathways enriched in DEGs.

We utilized the RNA-seq data of stomach adenocarcinoma samples from the TCGA data portal (https://portal.gdc.cancer.gov/) as the training set. Meanwhile, the available clinical data were also collected, including age, gender, pathological T stage, pathological N stage, pathological M stage, pathological TNM stage, grade, tumor location, the number of examined lymph nodes (ELNs), and the number of positive lymph nodes (PLNs). Of note, to improve the accuracy and robustness of screening LNM-related genes, samples with the following conditions were excluded: (1) information of pathologic T/N/M stage was not reported or with distant metastasis; (2) ELNs or PLNs were not mentioned; (3) with N0 stage disease but the number of ELNs was less than 16; (4) age unknown; (5) accepted neoadjuvant therapy. Finally, a total of 242 samples were enrolled for differentially expressed genes (DEGs) analysis (Fig. 1A).

As a validation set, log_10_-transformed gene expression data of 300 GC patients were downloaded from the ACRG dataset (GSE62254), which included 38 stage N0 cases and 262 samples with LNM [14].

### 2.2 Establishment and validation of LNM-related gene signatures

DEGs between pN0 and pN + samples were identified using package *edgeR* in R software. We chose a cutoff value of |log_2_ fold change| > 1 and a false discovery rate (FDR) < 0.001. Subsequently, 547 genes were identified, and the volcano plot was plotted by performing the R package *ggplot2*. Gene Ontology (GO) and Kyoto Encyclopedia of Genes and Genomes (KEGG) enrichment analyses were conducted and visualized by using the R package *clusterProfiler*. *P*-values <0.05 were considered statistically significant for GO terms and KEGG pathways.

Next, the normalized and log_2_-transformed expression data obtained by the *DEGList* and *calcNormFactors* functions in the *edgeR* package were used to construct the gene signature. We initially excluded the genes not expressed in 50% of the samples and not retrieved in the ACRG cohort to improve the clinical applicability of the proposed gene signature. Hence, 322 genes were selected for univariate logistic regression analysis; then, those found to be statistically significant were subjected to the least absolute shrinkage and selection operator (LASSO) regression analysis by the R package *glmnet* [15]. In our study, 10-fold cross-validation was performed for tuning parameter selection. Finally, thirty-three genes were selected to build the risk signature based on the optimal lambda value and the coefficients (Fig. S1). Risk scores of GC samples from the TCGA (n = 228) and ACRG (n = 300) cohort were calculated with the following formula: risk score = sum of LASSO coefficient of Gene Gi × expression value of Gene Gi [7]. Thus, the patients were dichotomized into groups of high- or low-risk by the median risk score.

To examine the performance of this gene signature in predicting LNM, we conducted univariate and multivariate logistic analyses. In addition, the discriminatory power of the gene signature and clinical variables was estimated by the area under the curve (AUC) for the receiver-operating characteristic (ROC) curve using the R package *pROC*.

### 2.3 Survival data and survival analysis

We downloaded the disease-free survival (DFS) and overall survival (OS) data of the TCGA cohort from cBioPortal (https://www.cbioportal.org). And the survival data for the ACRG cohort were obtained [14]. The survival data are summarized in Supplementary Table 1. The 33-gene risk score and clinical variables were applied to the survival analysis. Cox proportional hazards model and Kaplan-Meier analyses were performed using the R package *survival* and *survminer*.

### 2.4 Statistical analysis

Statistical analyses were performed using R software (version 4.1.2). Hazard ratios (HR) and 95% confidence intervals (95% CI) were calculated using the Cox proportional hazards model, and the odds ratio (OR) was obtained from the logistic regression analysis. The Wald test and log-rank test were conducted for the Cox model and the Kaplan-Meier analysis, respectively. Student's t-tests or one-way analysis of variance tests were used for continuous variables. All tests were two-tailed, and p-values <0.05 were considered statistically significant.

## 3 Results

### 3.1 Identification and enrichment analysis of LNM-related DEGs

We utilized the RNA-seq data of 242 GC samples consisting of 42 pN0 and 200 pN + patients from the TCGA cohort to identify LNM-related DEGs (Fig. 1A). Ultimately, compared with pN0 patients, a total of 547 DEGs were found, including 130 upregulated and 417 downregulated (Fig. 1B; Supplementary Tab. 2).

The GO functional enrichment analyses of DEGs were divided into three parts: biological process (BP), cellular component (CC), and molecular function (MF) [16] (Fig. 1C; Supplementary Tab. 3). We found the DEGs are significantly enriched in biological processes such as humoral immune response, epidermic development, skin development, and killing responses. In addition, they were particularly involved in the cellular component of the collagen-containing extracellular matrix. Concerning MF, these DEGs prefer receptor-ligand activity and signaling receptor activator activity. KEGG analysis highlighted the pathways associated with DEGs, such as the complement and coagulation cascades and the cholesterol metabolism (Fig. 1D).

### 3.2 Establishment and validation of the 33-gene risk score

**Fig. 2:**
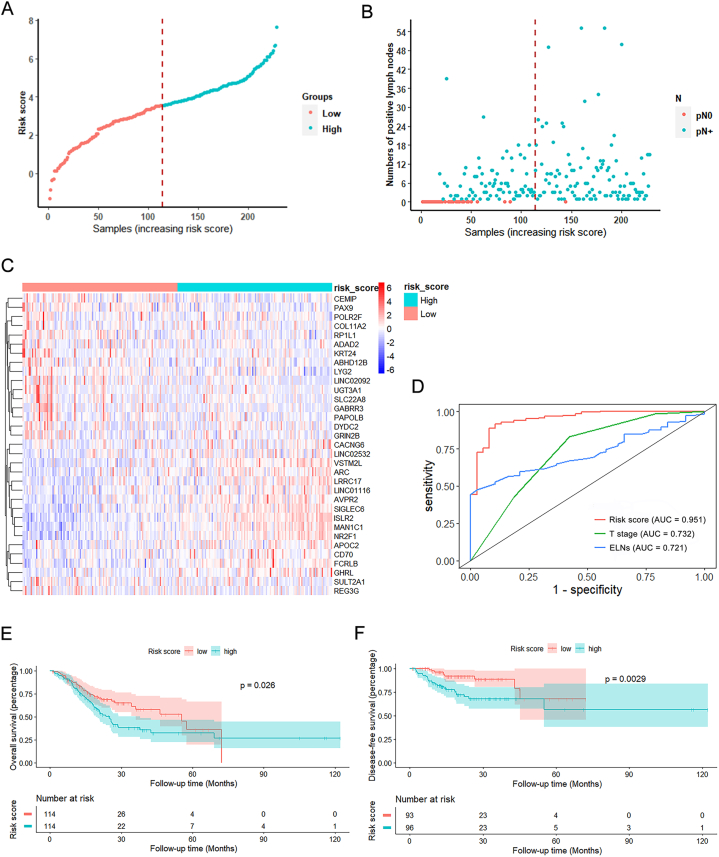
Characteristics and predictive value of the 33-gene risk score in the TCGA cohort. **A** The distribution of risk scores of 228 GC samples and the samples were divided into high- and low-risk groups by the median score. **B** The dot plot shows the lymph node status in high- and low-risk GC samples. The dotted line is the optimal cut-off value for dividing GC patients. **C** Heatmap representation of the expression level of 33 genes in 228 GC samples. **D** ROC curve analysis of the 33-gene risk score for predicting lymph node metastasis. **E-F** Kaplan-Meier survival curve shows the disease-free (**E**) and overall (**F**) survival rates between the high- and low-risk groups.

We initially planned to use the same sample pool for LNM-related gene signature identification and survival analysis; therefore, 228 GC samples with complete follow-up data from the TCGA cohort were enrolled (Fig. 1A). Then, we used a two-step screening method to identify a limited number of genes whose expression profile is significantly correlated with LNM of GC (Figure S1). First, univariate logistic regression analyses screened 161 genes associated with LNM (P < 0.05; Supplementary Tab. 4). Subsequently, LASSO logistic regression analysis identified 33 genes which were then applied to construct the LNM-related gene signature (Figure S2A, B; Supplementary Tab. 4). Besides, 33-gene based risk scores for these GC samples were calculated, and samples were then dichotomized into a high- and low-risk group using the median risk score (3.55) as the threshold value (Fig. 2A). As shown in Fig. 2B, the number of pN0 patients was significantly lower in the high-risk group. In addition, we used a heatmap to present the differentially expressed levels of these 33 genes in the high- and low-risk groups (Fig. 2C; Supplementary Tab. 5).

To validate the efficacy of this gene signature in differentiating pN+ from pN0 samples, we performed univariate logistic regression analyses. As expected, the 33-gene risk score was found to be associated with LNM (OR = 54.30, 95% CI = 7.30–404.14, *P* < 0.001), as were the following clinical variables: T stage, age, and ELNs (Table 1A). Moreover, multivariate logistic regression analysis indicated that only T stage (T3 and T4), ELNs, and the 33-gene risk score (OR = 52.84, 95% CI = 10.15–984.80, *P* < 0.001) are independent factors to predict metastatic lymphadenopathy (Table 1A). We then conducted ROC analyses to evaluate the predictive performance of the 33-gene risk score and compare it with clinical parameters that are significant in the logistic analysis. As shown in Fig. 2D, the risk score had the highest performance in the LNM prediction (AUC = 0.951, *P* < 0.001). Therefore, the 33-gene risk score seems to possess a superior performance to the classical clinicopathological features in predicting LNM.

**Table 1:** Risk factors for lymph node metastasis in GC. Univariate and multivariate logistic analyses were conducted to reveal the relationship of clinical variables and risk score with LNM in the TCGA (**A**) and ACRG cohort (**B**).

Variables	Logistic analysis in TCGA			
	Univariate analysis		Multivariate analysis	
	OR (95% CI)	*p*-value	OR (95% CI)	*p*-value
Risk score (high vs. low)	54.30 (7.30–404.14)	**< 0.001**	52.84 (10.15–984.80)	**< 0.001**
Age (≥65 vs. < 65 years)	0.41 (0.19–0.88)	**0.023**	0.57 (0.20–1.50)	0.262
Gender (male vs. female)	1.15 (0.56–2.37)	0.708		
ELNs	0.97 (0.96–0.99)	**0.002**	0.97 (0.95–0.99)	**0.031**
T stage (T1)				
T2	5.52 (1.27–24.08)	**0.023**	4.43 (0.73–43.32)	0.774
T3	22.81 (5.11–101.82)	**< 0.001**	32.09 (5.51–320.07)	**< 0.001**
T4	30.86 (6.65–143.25)	**< 0.001**	24.73 (4.16–246.35)	**< 0.001**
Grade (G1)				
G2	2.35 (0.36–15.24)	0.369		
G3	4.59 (0.72–29.39)	0.107		
Gx	2.00 (0.11–35.81)	0.638		
Location (cardia)				
Non-cardia	1.40 (0.65–3.01)	0.391		
Stomach, NOS	0.49 (0.08–3.00)	0.440		

Variables	Logistic analysis in ACRG			
	Univariate analysis		Multivariate analysis	
	OR (95% CI)	*p*-value	OR (95% CI)	*p*-value
Risk score (high vs. low)	2.41 (1.17–4.98)	**0.017**	2.34 (1.06–5.55)	**0.042**
Age (≥65 vs. < 65 years)	0.91 (0.46–1.80)	0.783		
Gender (male vs. female)	0.90 (0.43–1.86)	0.771		
ELNs	1.00 (0.98–1.02)	0.835		
T stage (T1/T2)				
T3	3.40 (1.27–9.05)	**0.015**	2.71 (1.05–8.45)	0.055
T4	1.88 (0.42–8.47)	0.413	1.61 (0.41–10.82)	0.547
Location (cardia)				
Body	0.35 (0.04–2.88)	0.330	0.34 (0.02–1.92)	0.312
Antrum	0.16 (0.02–1.23)	0.078	0.17 (0.01–0.89)	0.093
Entire	0.06 (0.01–0.88)	**0.040**	0.06 (0.002–0.81)	**0.039**

### 3.3 The 33-gene risk score associated with aggressive GC features

We performed Student's t-tests or one-way analysis of variance tests to analyze the distribution of the risk score. As shown in Table 2, a greater risk score was associated with aggressive tumor characteristics, including positive nodal metastasis (*P* < 0.001), deeper infiltration depth (*P* < 0.001), advanced TNM stage (*P* < 0.001), and lower grade of differentiation (*P* = 0.005). However, the risk score could not differentiate between N1, N2, or N3. (Table 2).

**Table 2:** Correlations between the 33-gene risk score and clinical parameters.

Variables	Distribution of the 35-gene score	
	Mean (±SD)	*p*-value^a^
Age (years)		**0.009**
≥65	3.13 (±1.52)	
<65	3.69 (±1.65)	
Gender		0.060
female	3.23 (±1.61)	
male	3.64 (±1.55)	
N stage		**< 0.001**
N0	1.04 (±1.09)	
N1	3.83 (±1.38)	
N2	3.82 (±1.23)	
N3	3.85 (±1.14)	
N1-3	3.83 (±1.24)	
T stage		**< 0.001**
T1	1.28 (±1.61)	
T2	3.18 (±1.94)	
T3	3.42 (±1.29)	
T4	3.68 (±1.26)	
TNM stage		**< 0.001**
I	1.01 (±1.32)	
II	3.30 (±1.70)	
III	3.79 (±1.21)	
Grade		**0.005**
G1	2.09 (±1.09)	
G2	3.01 (±1.54)	
G3	3.63 (±1.57)	
Gx	2.73 (±2.51)	
Location		0.590
Cardia	3.36 (±1.86)	
Non-cardia	3.42 (±1.48)	
Stomach, NOS	2.19 (±1.66)	

a *P* values for Student's t-test (two-tailed) or one-way analysis of variance test. The significant results are in bold.

### 3.4 Disease-free and overall survival analysis according to the risk score

First, survival analysis for patients from the 228 GC sample pool suggested that the high-risk group is associated with poorer OS (n = 228, *P* = 0.026 for the log-rank test; Fig. 2E) but not with DFS (n = 142, *P* = 0.490 for the log-rank test; Fig. S3). However, when we analyzed the DFS data of 189 patients from the whole TCGA cohort (Fig. 1A), the DFS rate was significantly worse in the high-risk group than in the low-risk group (*P* = 0.0029 for the log-rank test; Fig. 2F). In addition, Cox regression analyses of the 189 patients also indicated the risk score was an independent predictor of DFS (HR = 3.51, 95% CI = 1.53–8.06, *P* = 0.002; Table 3A).

**Table 3:** Association of the risk score and clinical variables with disease-free survival in GC. Univariate and multivariate logistic analyses were performed in the TCGA (**A**) and ACRG cohorts (**B**).

(A)				
Variables	Disease-free survival analysis in TCGA			
	Univariate analysis		Multivariate analysis	
	HR (95% CI)	*p*-value	HR (95% CI)	*p*-value
Risk score (high vs. low)	3.01 (1.40–6.45)	**0.005**	3.51 (1.53–8.06)	**0.002**
Age (≥65 vs. < 65 years)	1.11 (0.57–2.19)	0.755	1.31 (0.64–2.68)	0.444
Gender (male vs. female)	2.06 (0.96–4.43)	0.064	2.64 (1.19–5.85)	**0.016**
T stage (T1 + T2)				
T3 + T4	1.30 (0.56–3.02)	0.535	0.96 (0.19–4.81)	0.969
N stage (N0)				
N1	0.60 (0.21–1.66)	0.324	0.58 (0.17–1.96)	0.381
N2	1.06 (0.45–2.53)	0.893	1.43 (0.27–7.51)	0.665
N3	0.91 (0.37–2.27)	0.843	1.00 (0.17–5.80)	0.998
TNM stage (I)				
II	3.06 (0.87–10.73)	0.081	2.83 (0.35–22.53)	0.324
III	1.83 (0.52–6.41)	0.348	1.47 (0.06–31.67)	0.803
Location (cardia)				
Non-cardia	0.87 (0.38–2.02)	0.752	0.94 (0.39–2.22)	0.888
Stomach, NOS	2.72 (0.55–13.51)	0.222	2.50 (0.46–13.55)	0.288
(B)				
Variables	Disease-free survival analysis in ACRG			
	Univariate analysis		Multivariate analysis	
	HR (95% CI)	*p*-value	HR (95% CI)	*p*-value
Risk score (high vs. low)	1.87 (1.30–2.69)	**< 0.001**	1.51 (1.02–2.22)	**0.039**
Age (≥65 vs. < 65 years)	1.22 (0.86–1.75)	0.265		
Gender (male vs. female)	0.97 (0.67–1.40)	0.860		
ELNs	0.99 (0.98–1.00)	0.185		
T stage (T1/T2)				
T3	2.48 (1.70–3.60)	**< 0.001**	1.48 (0.92–2.37)	0.107
T4	2.64 (1.49–4.67)	**0.001**	0.72 (0.35–1.46)	0.359
N stage (N0)				
N1	1.75 (0.78–3.91)	0.175	1.30 (0.44–3.83)	0.639
N2	3.93 (1.76–8.76)	**0.001**	2.15 (0.68–6.73)	0.191
N3	7.37 (3.27–16.63)	**<0.001**	1.23 (0.40–3.74)	0.716
TNM stage (I)				
II	2.31 (0.69–7.74)	0.173	1.73 (0.36–8.41)	0.496
III	5.58 (1.73–17.97)	**0.004**	2.24 (0.42–12.00)	0.345
IV	13.57 (4.23–43.53)	**< 0.001**	8.80 (1.72–45.07)	**0.009**
Location (cardia)				
Body	0.75 (0.42–1.32)	0.315		
Antrum	0.75 (0.42–1.32)	0.107		
Entire	1.35 (0.45–4.06)	0.590		

### 3.5 External validation of the 33-gene risk score in the ACRG cohort

**Fig. 3:**
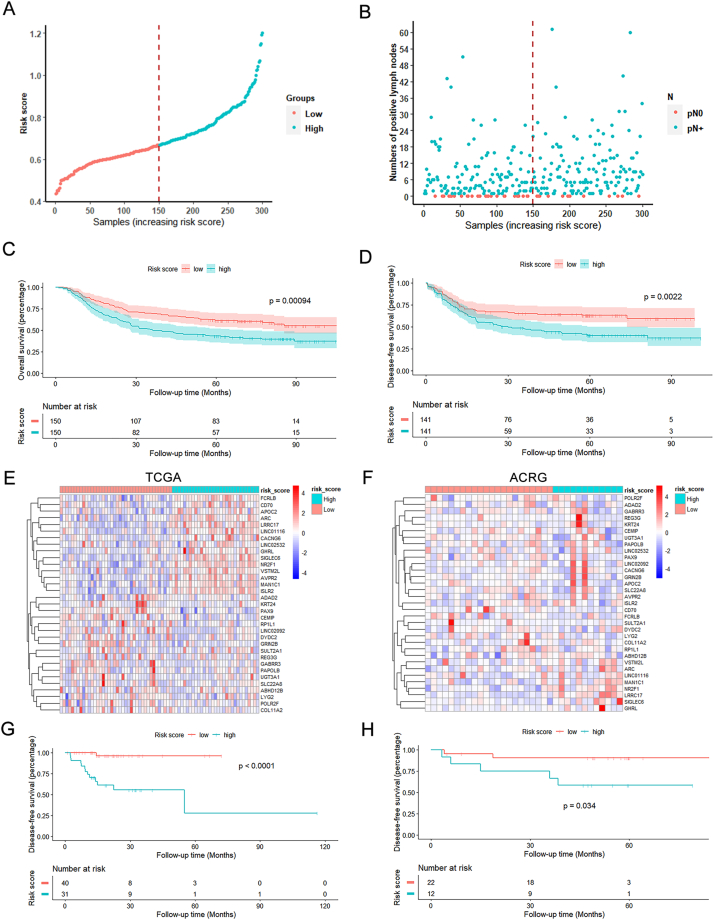
External validation of the 33-gene risk score in the ACRG cohort. **A** The dot plot shows the distribution of risk scores of 300 GC samples. **B** The distribution of lymph node status. **C**-**D** Overall survival (**C**) and Disease-free survival (**D**) analysis for the high- and low-risk group in the ACRG cohort. **E-F** Heatmap representation of the expression level of 33 genes in pN0 samples from the TCGA (**E**) and ACRG (**F**) cohort. **G-H** Disease-free survival curves were obtained by the Kaplan-Meier method in pN0 patients from the TCGA cohort (**G**) and the ACRG cohort (**H**), respectively.

The ACRG cohort was introduced as a validation group in this study. The coefficients derived from the TCGA cohort were directly applied, and patients in the ACRG cohort were then stratified by the threshold value of median score (0.668; Fig. 3A). As shown in Fig. 3B, the nodal status of patients was arranged by the risk score, and there were more pN0 patients in the low-risk group than in the high-risk group (26 vs. 12). Univariate logistic analyses revealed that the risk score (OR = 2.41, 95% CI = 1.17–4.98, *P* = 0.017) and T3 stage are risk factors for LNM; moreover, the multivariate logistic analysis showed the risk score (OR = 2.34, 95% CI = 1.06–5.55, *P* = 0.042) and tumor located in the entire stomach were independent factors (Table 1B). Importantly, the Kaplan-Meier analysis of OS revealed a worse prognosis for patients with a higher risk score (*P* < 0.001 for the log-rank test; Fig. 3C). Meanwhile, the high-risk group also correlated with poorer DFS (*P* = 0.002 for the log-rank test; Fig. 3D). Consistently, the multivariate COX regression analysis suggested that the risk score was an independent risk factor for DFS (HR = 1.51, 95% CI = 1.02–2.22, *P* = 0.039; Table 3B).

### 3.6 DFS analysis for the 33-gene risk score in pN0 patients

Several studies suggested that gene signatures could predict occult disease [17,18]. To explore the possibility of this 33-gene risk score in detecting tumor micrometastasis, we conducted DFS analysis for the 33-gene risk score in pN0 patients. Expression level of these 33 genes in pN0 samples were shown by heatmaps (Fig. 3E and F). It showed the survival was significantly worse in the high-risk group compared with low-risk cases in the TCGA cohort (*P* < 0.001 for the log-rank test; Fig. 3G). Similarly, survival analysis of stage N0 patients in the ACRG cohort also revealed a worse survival for the high-risk group (*P* = 0.034 for the log-rank test; Fig. 3H). Thus, this 33-gene risk score could identify a subgroup of patients with a worse outcome within the pN0 patients.

## 4 Discussion

Treatment options for GC depend on regional lymph node status and depth of invasion. In general, tumors confined to the mucosa with a negligible risk of developing LNM are better candidates for endoscopic resection; otherwise, gastrectomy with lymph node dissection is required. Meanwhile, lymph node-positive patients usually receive adjuvant chemotherapy because of the high probability of recurrence. Therefore, the identification of lymph node metastases is a critical part of the long-term management of patients with GC [1]. Molecular biological techniques such as immunohistochemistry or reverse transcription-PCR have been performed to improve the sensitivity of LNM detection in harvested lymph nodes, highlighting the value of molecular diagnostics for classifying lymph node status [19,20]. In addition, analysis of gene expression of primary tumors has also been proven useful for LNM detection in several cancers [8,21]. In GC, Li et al. using the gene expression data of primary tumors from the TCGA stomach adenocarcinoma cohort identified a 4-mRNA signature that could predict LNM [13]; however, it possesses some limitations that we had resolved in our study. For example, we excluded pN0 patients with ELNs less than 16 and included ELNs as a variable in the subsequent analysis to remove the impact of ELNs on N stage migration [22]. We also identified a 33-gene based signature using LASSO regression analysis which performs both feature selection and classification to improve the accuracy. In addition, we conducted DFS analyses to reveal the value of this LNM-related gene signature in predicting recurrence, and the obtained results were further validated in an independent cohort. Thus, with comprehensive design and rigorous statistics, our study defined a 33-gene based signature that could predict the lymph node status and future recurrence in GC.

Lymph nodes are critical for initiating antitumor immune responses [23]. GO functional enrichment analysis suggested that the DEGs were related to humoral immune response, acute inflammatory response, and several killing responses, which proved that cancer cells in the primary site interact with regional lymph nodes [24]. Meanwhile, cancer cells metastasize to lymph nodes involving the remodeling of the extracellular matrix; thus, the DEGs were also enriched in the cellular component of the collagen-containing extracellular matrix [25]. Another essential function of the lymphatic system is the transportation of dietary lipids. Hence, the enrichment of DEGs in cholesterol metabolism may imply the change in lipids metabolism in cancer cells because of the impaired function of the tumor-invaded lymphatic system [26]. Therefore, these LNM-related genes probably reflect the biological pathways attributing to the nodal metastatic phenotype. However, the detailed mechanism of how these DEGs involve in LNM demands further research.

Intriguingly, when we conducted a DFS analysis of 142 patients from the 228 GC sample pool, it showed no significant results. However, when we added 47 samples (including 26 pN0 patients with ELNs less than 16) into the survival analysis, the DFS rate was significantly different between the high- and low-risk groups. In addition, survival analyses of the 33-gene risk score in pN0 patients suggested a worse DFS for the high-risk group. Thus, these results remind us that a high-risk score may be a reflection of occult or residual lymph node disease in pN0 patients, resulting in a poor prognosis. On the other hand, it indicates that insufficient numbers of examined lymph nodes may underestimate the nodal stage and increase the chance of residual malignancy.

Gene expression signatures have been developed to improve tumor staging and to provide outcome predictors [27,28]. Examples of applications of our 33 gene-based signature include improving the assessment of the risk of LNM in early gastric cancer, improving the evaluation of tumor staging in patients with both inadequate lymphadenectomy and negative LNM, strengthening the ability to predict the risk of postoperative recurrence, and identifying biological targets for the development of new drugs. However, how to transfer this signature from the bench to the bedside? Tissue samples can be obtained from GC patients by preoperative gastroscopy or gastrectomy. Then the expression of these 33 genes could be detected by a PCR array while a risk score is obtained to assess the risk of LNM and recurrence. Of note, such a risk model needs to be prospectively validated in a larger sample size, and integrating gene expression and pathological features for assessment may be a better approach.

Although this 33-gene based signature had good performance in predicting LNM and prognosis, there existed some limitations. First, factors like lifestyle (such as smoking history, drinking history, and diet) were not included, which can impact the clinical response of patients [29]. Second, the absence of survival data in some cases made it impossible to guarantee the consistency of sample sizes in the TCGA cohort. Third, this gene signature could not discriminate between N1, N2, or N3. Fourth, although with the validation in the ACRG cohort, the expression of these 33 genes was not reevaluated in GC tissues by techniques such as quantitative real-time PCR. Fifth, the specific mechanism of these genes involved in LNM of GC was not further analyzed.

In summary, we developed a 33-gene based signature that could predict lymph node status and prognosis in patients with GC. Furthermore, this 33-gene risk score indicates a subgroup of pN0 patients prone to future recurrence. However, future studies are warranted to validate our findings.

## Author contribution

Jian Xiao; Gang Wang; Chuming Zhu: Conceived and designed the experiments; Performed the experiments; Analyzed and interpreted the data; Contributed reagents, materials, analysis tools or data; Wrote the paper.

Kanghui Liu; Yuanhang Wang; Kuan Shen; Hao Fan; Xiang Ma: Analyzed and interpreted the data; Contributed reagents, materials, analysis tools or data.

Zekuan Xu; Li Yang: Conceived and designed the experiments; Performed the experiments; Wrote the paper.

## Funding

This study was supported by the 10.13039/501100001809National Natural Science Foundation of China (Grant No. 81874219) and the Project of Cultivating Innovation in Science and Technology Plan of Liyang City (Grant No. LC2021001).

## Data availability statement

The data could be downloaded at (https://portal.gdc.cancer.gov/, https://www.cbioportal.org, and https://www.ncbi.nlm.nih.gov/geo/; GSE62254) and the code used during the current study are available from the corresponding author on reasonable request.

## Ethical approval

The authors are accountable for all aspects of the work in ensuring that questions related to the accuracy or integrity of any part of the work are appropriately investigated and resolved. There was no need for ethical approval as all data in this study were downloaded from public databases, and the data processing met the publication guidelines.

## Declaration of competing interest

The authors declare that they have no known competing financial interests or personal relationships that could have appeared to influence the work reported in this paper.
