# Supplementary material for: A thirty-three gene-based signature predicts lymph node metastasis and prognosis in patients with gastric cancer
Source: Heliyon. 2023 Jun 5;9(6):e17017. doi: 10.1016/j.heliyon.2023.e17017 (PMC10361117; doi:10.1016/j.heliyon.2023.e17017)
Supplement: Multimedia component 1 [file mmc1.pdf]

Title: A thirty-three gene-based signature predicts lymph node metastasis and prognosis in patients with gastric cancer

Author information

Jian Xiao[1, a], Gang Wang[1, a], Chuming Zhu[2, a], Kanghui Liu[1], Yuanhang Wang[1], Kuan Shen[1], Hao Fan[1], Xiang Ma[1], Zekuan Xu[1], Li Yang[1, 2]

[1] Department of General Surgery, The First Affiliated Hospital of Nanjing Medical University, Nanjing, Jiangsu Province, China.

[2] Department of General Surgery, Liyang People's Hospital, Liyang Branch Hospital of Jiangsu Province Hospital, Liyang, Jiangsu Province, China.

[a] These authors contributed equally to this work.

Corresponding author: *Li Yang*

Supplementary Figures

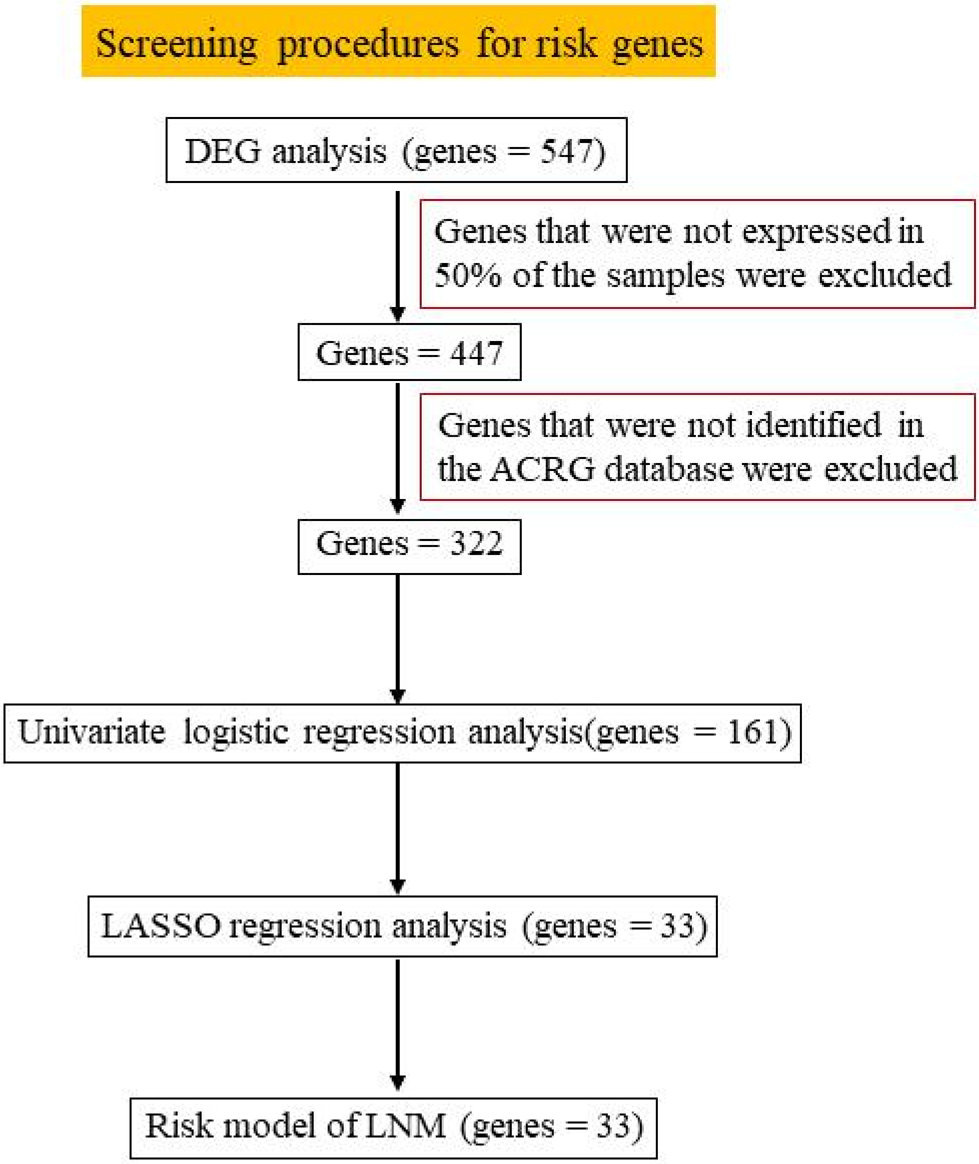

Figure S1 The flow chart shows the procedures for the establishment of the 33-gene based signature.

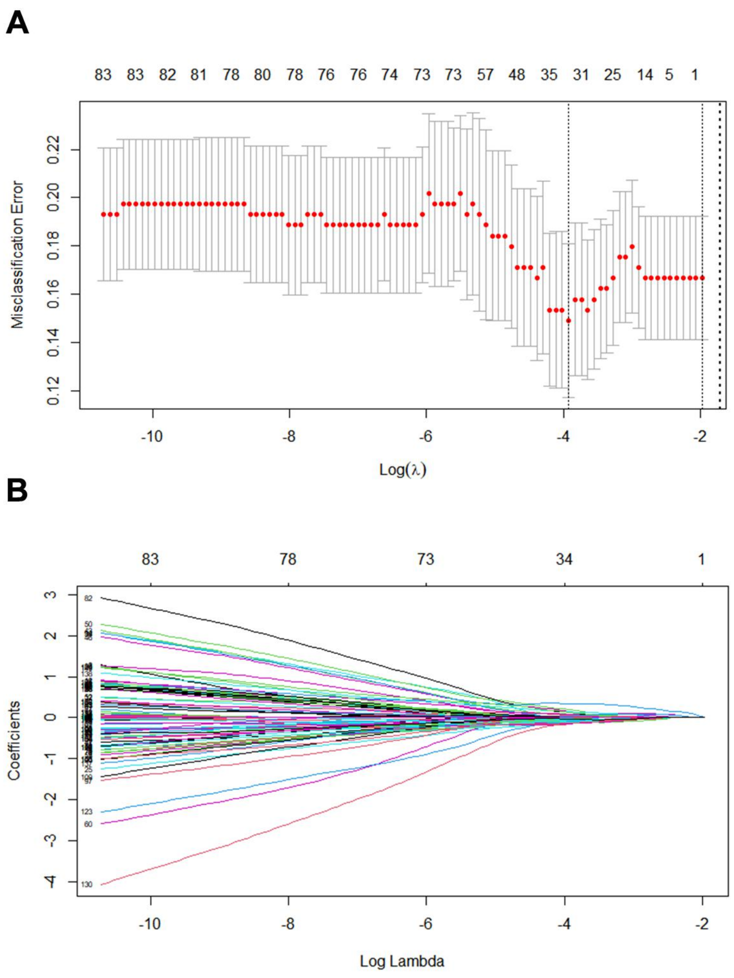

Figure S2 LASSO logistic regression analysis for 161 differentially expressed genes. (**A**) Thirty-three genes were selected according to the minimum value of lambda. (**B**) The coefficients of genes by LASSO logistic regression analysis.

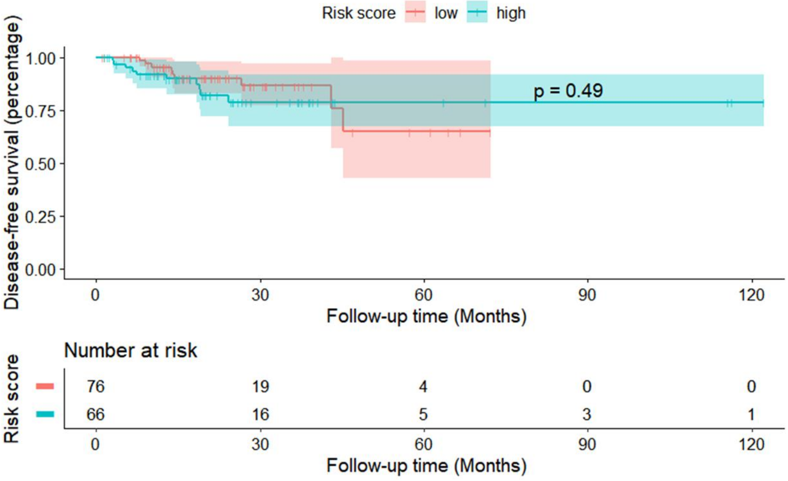

Figure S3 Disease-free survival analysis of the risk score in 142 GC samples.
